# Art education lecturers’ intention to continue using the blackboard during and after the COVID-19 pandemic: An empirical investigation into the UTAUT and TAM model

**DOI:** 10.3389/fpsyg.2022.944335

**Published:** 2022-10-03

**Authors:** Abeer S. Almogren

**Affiliations:** Department of Art Education, College of Education, King Saud University, Riyadh, Saudi Arabia

**Keywords:** blackboard, art education, UTAUT, structural equation modeling (SEM), learning and teaching (L&T), COVID-19 pandemic

## Abstract

The success of faculty in adopting technology in this digital era has a direct impact on the success of the students and, eventually, the educational institution. Many teachers, on the other hand, have yet to implement technological tools such as Canvas into their classes. As a result, this study looked at art universities lecturers’ opinions of variables influencing actual blackboard use, as well as their desire to utilize the lesson plan in learning and teaching during the COVID-19 Pandemic. The TAM model and the unified theory of acceptance and use of technology (UTAUT) were used to analyze lecturers’ satisfaction with the blackboard and their desire to continue using it, as well as the actual usage of blackboards. A survey of 159 professors using Canvas in art instruction at five Saudi Arabian public institutions yielded the research findings. In addition, structural equation modeling (SEM) was used to evaluate the study model as well as the mediating relationship between factors influencing the desire to implement the lesson in learning and teaching during the COVID-19 Pandemic. According to the study’s findings, superior impact, performance expectation, effort expectations, enabling conditions, and reported enjoyment all had statistically significant effects on perceived usefulness (PU) and perceived ease of use. The current study discovered that PU and perception of use had statistically significant influence on behavior intent, actual blackboard usage, and lecturers’ happiness with utilizing blackboard in learning and teaching during the COVID-19 Pandemic. Furthermore, the results demonstrate that lecturer’s intention to continue utilizing chalkboard in learning and teaching during and after COVID-19 Pandemic was influenced by actual usage of blackboard and reported pleasure. The findings are useful for education based, regulators, and practitioners who seek to create and enhance effective methods to use e-learning systems during and after the COVID-19 Pandemic.

## Introduction

Information technology (IT) and information systems (IS) have had a significant influence on the strategic management of the learning ecosystem at higher learning institutions in the twenty-first century (Singh and Rathod, 2019) through the use of appropriate types of learning platforms (Attuquayefio, 2022). As a result, each education institution’s choice of a suitable learning management system (LMS), such as Canvas, is crucial (Alturki and Aldraiweesh, 2021). As a result, Blackboard is simply described as “web-based technology that aids in the development, delivery, and assessment of a particular learning process” (Ibrahim et al., 2022; Tamada et al., 2022). Indeed, information and communication technologies (ICTs) have had a massive and cumulative influence in the academic environment, boosting the manageability of learning procedures more adequately and efficiently (Al-Rahmi et al., 2020). A Blackboard clearly facilitates and supports administration and technological capabilities that are tailored rendering to educational process requirements for both educators and pupils (Cavus et al., 2021). As a result, a number of organizations have made significant investments in their systems in order to develop an experience and understanding society (Arshad et al., 2020). Furthermore, the Blackboard would most likely encourage greener behaviors by minimizing the amount of paper needed (Mohammadi et al., 2021). Blackboard provides professors with useful tools for managing course content in a flexible manner. It enables teachers to create electronic interactive courses and handle duties such as assignments, quizzes, scores, course overview, email sending and receiving, and student announcements. Professors can also split students into groups using the Blackboard system, which provides for immediate live-chat among group members with the option for professors to administer these live-chats. Furthermore, the system has a function that allows teachers to track the multitude of times students visit the system and use the materials posted on Blackboard, as well as generate reports on that information. Despite the benefits of Blackboard, many professors do not utilize it in their classes (Al Meajel and Sharadgah, 2018). Despite the fact that Blackboard was created to help long-distance learning users build a communication medium (Alyahya and Bhatti, 2022), it has recently become a popular tool for face-to-face education (Gamede et al., 2022; Gurban and Almogren, 2022). As a result, several studies have been undertaken to demonstrate the adoption and use of Blackboard at educational institutions that rely heavily on online or virtual learning methods (Almelhi, 2021; Al-khresheh, 2022; Alsalamat et al., 2022; Lavidas et al., 2022). In today’s environment, technology is widely used in education. As online technology develops and changes, such as learning management systems (LMSs) like Blackboard, we’re seeing more complex software systems designed to handle educational activities, with an emphasis on supporting teachers in imparting information to their students. Learning institutions can use Blackboard to store, manage, and share topic and course contents (Alhadreti, 2021). The COVID-19 epidemic has wreaked havoc on traditional learning and teaching methods (Mujalli et al., 2022) and sparked a significant global crisis in the way the higher education system operates (Karalis, 2020). Many elements impact online learning; instructors and students both feel engaged and rewarded when they attend classes electronically (Moawad, 2020). Professors and students at universities who utilize Blackboard, Zoom, or Google Class to develop learning systems may confront significant technological problems (Sawaftah and Aljeraiwi, 2018). When using online learning systems like Blackboard, poor infrastructure and a lack of technical support can make it difficult to offer good instruction. Blackboard has the largest market share in online teaching and learning systems (Sultana, 2020). In comparison to poor nations, blackboard is becoming more popular in industrialized countries. Blackboard has recently introduced a number of new possibilities and features.

### Problem background

At Saudi Arabia, the usage of Blackboard throughout public universities has recently gained popularity in comparison to traditional face-to-face education. As a result, the use of ICT tools such as Canvas (Ibrahim et al., 2019; AlKarani and Thobaity, 2020; Alturki and Aldraiweesh, 2021) is no longer restricted to long-distance education. However, just a few surveys have shown instructors’ satisfaction with Canvases in the context of traditional learning at Saudi state bodies, and also their intention to utilize it in the foreseeable. As a result, this research looks into the overall usefulness of the unified theory of acceptance and use of technology (UTAUT) in such circumstance. In reality, the success of information systems like Blackboard is strongly reliant on how well end users embrace and perceive their benefits (Bouznif, 2018; Alshehri et al., 2020; Sultana, 2020). This study focuses on art education at five Saudi Arabian universities’ faculties of education, with the aim of determining the usefulness of the UTAUT and TAM models in identifying lecturers’ pursuit of Blackboard usage in a face-to-face teaching and learning approach. Prior research has revealed that Blackboard usage at Saudi institutions is still in its early phases, in other words, it hasn’t been fully explored (Alduraywish et al., 2022). In this scenario, it appears that there is a disconnect between the advanced technological features supplied by LMS such as Blackboard and their effective use in Saudi institutions (Ahmad et al., 2021). According to Alturki and Aldraiweesh (2021), this gap may be traced back to a lack of strategic direction from university administration, especially if Blackboard is still in its early stages of implementation in Saudi Arabia. Saudi Arabian institutions, for example, have lately migrated from faculty websites to Blackboard for their courses. This drastic transition, especially in its early phases, entails a degree of resistance in lecturers’ perceptions of the amount of work necessary to be a good Blackboard user (Taylor and Todd, 1995; Venkatesh et al., 2003). According to Alduraywish et al. (2022) and Al-Mohair and Alwahaishi (2020), the level of user satisfaction might influence the deliberate behaviors of the user’s decision to continue or stop using something. As a result, universities must conduct a thorough study of the accessibility of online learning ahead of time. As previously indicated, as a result of the COVID-19 pandemic, there is a higher reliance on online education delivery technologies as an emergency response. The purpose of this article is to seek comments from Blackboard lecturers throughout COVID-19 and beyond. The study’s purpose is to identify and assess the factors that impacted arts degree lecturers’ and faculty members’ use of this platform during the COVID-19 epidemic, as well as to see if lecturers intend to use the blackboard once the pandemic is finished. Prior to the outbreak, the use of online learning platforms like Canvas was still in its early stages in Saudi colleges, which is why art at Saudi institutions was selected for this reason. There was a crisis as a consequence of the outbreak. Crises usually need quick responses and actions. Despite the fact that several higher education institutions around the world have needed urgent responses, such as a quick shift to e-learning, an environment like a Saudi university aids in the identifier of important effective factors that influence fast technological implementation, such as Chalkboard adoption. Lecturers and faculty inside the art education field were chosen because they generally teach large groups and because technology use in the discipline has lately been encouraged from an educational standpoint. This article’s main contribution is to assess user experience with the Blackboard system during and after the COVID-19 Pandemic (Vrielink, 2015). Study looked into the use of chalkboards, but his conclusion was limited to determining the behavioral intent of lecturers and students to use blackboards. There is a lack of research into other factors influencing students’ use, which will undoubtedly motivate researchers to look into this topic further. As a result, this study intends to address this vacuum by testing behavior intention, actual use along with instructors’ satisfaction, and continuous use intention using TAM’s aspects, namely perceived ease of use (PEU) and perceived utility. In addition, this article contributes to the UTAUT model by increasing our understanding of the influence of various factors on the use of Blackboard platforms. As a result, the purpose of this research was to develop a new methodology for assessing art education instructors’ true and continuing intentions to use the blackboard during and after the COVID-19 outbreak.

## Research theories and hypotheses development

**FIGURE 1:**
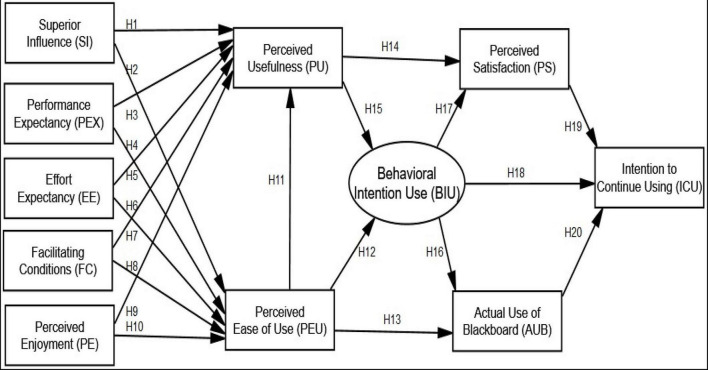
Research model and hypotheses.

The purpose of this research is to learn more about the key factors that impact art education instructors’ decision to keep using Blackboard after the COVID-19 outbreak. This research used two theories, TAM and UTAUT, to develop a model. These theories are preferred above other acceptance technology models because of their comprehensive, deep, and wide insights (Venkatesh et al., 2003; Lwoga and Komba, 2015). The development of TAM and extended TAM models in an e-Learning scenario was noted in a previous study in Saudi Arabia on lecturers’ and students’ plans to utilize Blackboard (Alharbi and Drew, 2014; Alshammari et al., 2016). According to Bellaaj et al. (2015) investigated students’ intentions about Blackboard usage at the University of Tabuk in Saudi Arabia, using UTAUT in the context of virtual learning. Therefore, this research integrated the UTAUT and TAM models, which were used to develop the research model for this study. During the COVID-19 epidemic, acknowledgment and use of technology methods were also used at King Saud University to investigate the impact of students’ interaction on learning as well as the use of a learning system (LMS), where TAM has surfaced as a highly promising tool for determining students’ actual intent to use cloud computing (Alturki and Aldraiweesh, 2021). As a result, only a few research have taken advantage of the value of the UTAUT paradigm in addressing the usability and acceptability of Blackboard in conventional education. According to Venkatesh et al. (2003), the UTAUT model consists of four fundamental elements (performance expectation, effort expectancy (EE), social influence, and enabling circumstances) that have significant implications for predicting user intention and behaviors related to technology adoption and usage. The second change is that satisfaction has been added to the UTAUT model, which some studies show is crucial in predicting whether students will continue to use Blackboard. Mouakket and Bettayeb (2015) and Ramayah and Lee (2012) shows how professors feel about utilizing Blackboard with their students (Taylor and Todd, 1995). As a result, this study created a new model based on the following variables: Greater Influence (SI), Finally, a minor adjustment can be made by decomposing (social influence) into (superior influence), which adequately accounts for Performance Expectancy (PE), EE, Facilitating Conditions (FC), Perceived Usefulness (PU), PEU, Perceived Enjoyment (PE), Behavioral Intention (BI), Actual Use of Blackboard (AU), Perceived Satisfaction (PS), and Original intent to Continue Using (see Figure 1).

### Performance expectancy

The amount to which instructors anticipate that using Blackboard would improve teaching and learning results by obtaining stunningly remarkable and desirable successes in their assignments is characterized as PE. Based on previous research, there is a strong link between performance expectations and PU, PEU with attitude toward usage, and actual use of LMS and chalkboard (Bellaaj et al., 2015; Alhadreti, 2021; Alturki and Aldraiweesh, 2021; Alyoussef and Al-Rahmi, 2022). Furthermore, PU in TAM has a direct effect on satisfaction (Cheok and Wong, 2015; Al-Rahmi et al., 2022). As a result, the more delighted lecturers are with the results obtained from utilizing Blackboard, the more likely they are to continue using it in the future (Calli et al., 2013). The following hypotheses were suggested based on the discussion above:

PE is positively associated with PU.

PE is positively associated with PU.

### Effort expectancy

The degree to which teachers feel a substantial amount of ease or difficulty in utilizing Blackboard is referred to as effort expectation. Clearly, two constructs had a role in the EE: “PEU” and “perceived utility” (Venkatesh et al., 2003; Raza et al., 2021). According to Yoo et al. (2012), the effort expectation, which is considered an intrinsic feature of the UTAUT model, is the amount of effort that individual expects to invest to use a technology, which is often minimal owing to the consumer nature of IoT technology (Dečman, 2015). Previous study has found a significant positive relationship between effort expectations and PEU and utility in turn in, which promotes long-term willingness to utilize LMS like Blackboard, particularly in the early phases of technology acceptance (Bellaaj et al., 2015; Alshehri et al., 2019). According to Cheok and Wong (2015) the usefulness and perceived simplicity of use in TAM is a key determinant of satisfaction. As a result, the more delighted lecturers are with the ease with which they may use Blackboard, the more likely they are to use it in the long run (Cao, 2022). The following hypotheses were suggested based on the discussion above:

EE is positively associated with PU.

EE is positively associated with PEU.

### Superior influence

The term “SI” refers to a lecturer’s powerful and targeted effect in motivating and inspiring other academics to use Blackboard. Social influence may be divided into three groups (Taylor and Todd, 1995): peer, superior, and subordinate. As a result, this study is more relevant to academics since it focuses entirely on SI. Indeed, social circumstances have an impact on SI, which is a component of social influence (Venkatesh et al., 2003). When Fidani and Idrizi (2012) investigated the factors impacting the field of e-learning systems, they observed that social influence had a positive impact on attitudes toward and actual use of LMS such as Blackboard. Previous research has established a positive relation between social influence and continued intention to use LMS such as Blackboard (Lwoga and Komba, 2015; Alturki and Aldraiweesh, 2021; Raza et al., 2021), while another study discovered a negative relationship (Bellaaj et al., 2015; Lwoga and Komba, 2015; Alturki and Aldraiweesh, 2021; Raza et al., 2021). This implies that lecturers’ judgments of superior impact are a consistent indicator of reported ease of use and perceived in turn in, as well as effect mood about Blackboard usage and long-term willingness to use it. The following hypotheses were suggested based on the discussion above:

SI is positively associated with PU.

SI is positively associated with PEU.

### Facilitating conditions

Providing conditions, according to Venkatesh et al. (2003), are the availability of enough resources and support for the proper application of technology. FC in the online teaching environment are defined as the availability of technological and organizational infrastructure enabling the adoption and usage of a learning management system (LMS) such as Blackboard. This involves education, technical assistance, and the necessary infrastructure (Dečman, 2015). According to Nanayakkara (2007), students’ acceptance of web-based technology will be hampered by a lack of assistance and continuous encouragement due to limited resource availability and information, because they rely on their teachers’ and technical support to positively affect their use of Blackboard (Ain et al., 2016). This suggests that lecturers’ perceptions of the availability of conducive conditions have an impact on their reported ease of use and usefulness in turn in, as well as their attitude toward using Blackboard and their continuous desire to utilize it. The following hypotheses were suggested based on the discussion above:

FC is positively associated with PU.

FC is positively associated with PEU.

### Perceived enjoyment

Perceived delight is a natural urge that describes how much pleasure may be received from using a system (Chao, 2019). It refers to the point at which utilizing a system appears to be pleasurable, regardless of how well the system performs (Park et al., 2012). Enjoyment is linked to perceived utility and simplicity of use, as well as the intention to utilize technology (Hanif et al., 2019; Sarosa, 2019). If a system is considered pleasurable, people are more likely to accept and use it. In various LMS, such as Blackboard (Sarrab et al., 2017), validated the importance of users’ delight. Previous research (Akhter et al., 2022; Şahin et al., 2022; Yaghi, 2022) has found that pleasure influences the perceived utility of Blackboard and online learning in Saudi Arabia (Al-Gahtani, 2016; Ibrahim et al., 2019; Khafaga, 2021). The following hypotheses were suggested based on the discussion above:

PE is positively associated with PU.

PE is positively associated with PU.

### Perceived usefulness and ease of use

The original TAM’s major factors are PU and PEU, which is defined as the degree to which students believe it will be simple to utilize the Blackboard during COVID-19 (Alturki and Aldraiweesh, 2021). Blackboard, as an online LMS, has a beneficial influence on academic achievement throughout the COVID-19 pandemic period (Chan and Zhang, 2021). The Technology Acceptance Model (TAM) was used to start investigating the use of blackboard by teachers and students in Dutch police universities, and it was discovered that lecturers’ behavior intention to adopt blackboard was substantially lower than students’, indicating that blackboard was more popular among students. Furthermore, Moonsamy and Govender (2018) discovered that several employees who were first trained on the chalkboard have now switched to other methods. Blackboard also mentioned that it is difficult to use and may not be user friendly. Although there have been studies on the use of chalkboards by teachers and students in the past, the use of blackboards in the university environment is still mostly among professors, necessitating additional study on the use of blackboards from the perspective of lecturers (Davis, 1989). Established the Technology Acceptance Model (TAM), which has been widely utilized in technology acceptance and has emerged as a major model for predicting user intention and adoption behavior. Individuals’ actual usage of the system is governed by behavioral intention, which is driven by perceived utility and PEU TAM’s model (Vrielink, 2015). In other words, the present study looks at how lecturers view the blackboard in terms of PEU and utility, as well as whether the blackboard system’s interaction is clear and what specific functions impact lecturers’ opinions of the blackboard as helpful and simple to use. The following hypotheses were suggested based on the discussion above:

PEU is positively associated with PU.

PEU is positively associated with BIU.

PEU is positively associated with AUB.

PU is positively associated with PS.

PU is positively associated with BIU.

### Behavioral intention of blackboard use

His or her intention refers to a person’s adoption intention the usage of a given technology for various activities (Ain et al., 2016; Zhao et al., 2021). Defined behavior intention as a person’s willingness to engage in a certain conduct, which in this article refers to lecturers’ willingness to embrace the usage of Blackboard to meet their academic course objectives. Several researchers have looked at the impact of technology’s behavioral intention on its actual usage behavior and discovered that there is a direct and substantial link (Davis, 1989; Motaghian et al., 2013; Unal and Uzun, 2021). In a publication, Nicholas-Omoregbe et al. (2017) found that the behavioral intention to adopt an online learning system had a positive relationship with actual use and sustained usage intention. The following hypotheses were suggested based on the discussion above:

BIU is positively associated with AUB.

BIU is positively associated with PS.

BIU is positively associated with ICU.

### Actual use of blackboard

Blackboard is a virtual education management system that helps teachers and students succeed. Audios, movies, PowerPoint, animation, links, and other learning resources can be added to Blackboard course content by the teacher (Al-Oqaily et al., 2022). Blackboard apps have the ability to change learners’ attitudes about learning and teachers’ teaching methods (Hakim, 2020). Based on data from earlier studies on the use of Blackboard as a teaching tool (Ja’ashan, 2015; Pusuluri et al., 2017; Alakrash and Razak, 2020), the study revealed the association between students’ perceptions and actual usage of Blackboard for learning purposes. To deal with difficult situations, educational institutions provided a thorough guide for students and teachers, as well as assistance in accessing online learning *via* Blackboard platforms (Yen, 2020). Universities in Saudi Arabia used the Blackboard platform as an online learning system that included all topographies and accommodations to create a comparable environment to that of actual classrooms (Ali, 2017; Basilaia et al., 2020). Also conducted a multi-method investigation of students’ perceptions of Blackboard as an online learning tool. Other research (Elfaki et al., 2019; Gördeslioğlu and Yüzer, 2019; Alamer, 2020) found substantial links between students’ perceptions and usage of Blackboard as a learning tool (Alamer, 2020). Evaluated King Khaled University students’ perceptions regarding the usage of Blackboard as a learning tool. The following hypotheses were suggested based on the discussion above:

AUB is positively associated with ICU.

### Perceived satisfaction

Satisfaction is an emotional evaluation of various outcomes that may also be used to beautiful or disturbing opinions. Satisfaction is a useful variable in studies of online support (Chan et al., 2010) and the effectiveness of online administration since it reflects common attitudes based on previous contacts with online services (Oliver, 1980). In addition, (Mazrou et al., 2013; Al-Sadhan, 2015; Almoeather, 2020), investigated the views of university students and faculty members about the Blackboard system. Learner satisfaction, behavioral control, and the efficiency of the Canvas learning system were examined by Liaw (2008). Usefulness and gratification both contribute to learners’ behavioral intention to use the Lesson plan, and it’s a crucial component that determines learners’ contentment with the Blackboard learning system, according to the findings. Instructional material, active learning activities, and the quality of the Blackboard learning system can further impact the efficacy of online learning. Other studies were carried out to see how beneficial Blackboard was thought to be (Nguyen, 2021; Saxena et al., 2021). According to Limayem and Cheung (2008), PU had a substantial impact on satisfaction and desire to continue using the Lesson plan of all first business students at one university (Lee, 2010b). Discovered that PEU and usefulness effects satisfaction and continuation intention among students who are given e-learning services in National Pingtung University’s continuing education program in Taiwan. The following hypotheses were suggested based on the discussion above:

PS is positively associated with ICU.

### Intention to continue using blackboard

The willingness to use an information system (IS) in the future and to suggest it to others is referred to as continuation intention (Chang, 2013). According to the expectation confirmation model, user happiness is the most essential component in determining a user’s desire to continue using a product (Bhattacherjee, 2001; Rahi et al., 2018; Foroughi et al., 2019; Huang, 2019). “Satisfaction, is an assessment of feeling related with an emotional attitude toward a system” (i.e., whether the utilization experience is pleasurable as expected) (Sabah, 2019). As a result, a user may have a good attitude toward a system but yet be unhappy after using it owing to low expectations, which is the same notion as the goal to utilize technology indefinitely (Tran et al., 2019). Users will have a sense of pleasure regarding a specific technology (Cheng, 2014; Alraimi et al., 2015), and will have the desire to continue using it if they find it beneficial. In several technological contexts, previous research has established the important association between satisfaction and continued intention (Alraimi et al., 2015; Foroughi et al., 2019; Sabah, 2019; Alshurideh et al., 2020). Previous research has discovered that the degree to which users consider an information system to be useful has a favorable impact on their desire to use it again (Bhattacherjee, 2001; Hoehle et al., 2011). Prior research has revealed that PU has a major impact on university students’ propensity to use Blackboard in the long run (Limayem and Cheung, 2008; Lee, 2010a).

## Research methodology

The data were then analyzed using the Statistical Package for the Social Sciences (SPSS) and structural equation modeling (PLS-SEM) to validate the validity and reliability of the measurement model. This study was conducted online from February to April 2022, distributed 237 questionnaires randomly as part of the analysis to university staff. However, 21 participants’ responses contained incomplete answers and were not included in the analysis. A total of 15 participants’ responses were missing data and 6 participants’ responses were removed after the normality test. Such exclusions were suggested by Hair et al. (2019) and Ringle et al. (2005), who claimed that outliers would lead to incorrect statistical results and should be omitted. As a result, 195 participants’ responses were imported into the SPSS program. The most common quantitative research approach for data collecting is surveys, which are common in social science research. A five-point Likert scale questionnaire was used in this study as a quantitative data gathering instrument (Jamieson, 2004). Each question is a statement to which the responder must assign a number between 1 and 5 to indicate how strongly they agree or disagree with it (for example, 1 = strongly agree, 2 = agree, 3 = neither agree nor disagree, 4 = disagree, 5 = strongly disagree). Performance expectation, EE, SI, enabling situations, and reported enjoyment were all investigated separately. PU and PEU are mediated by two elements. Behavioral intention, actual usage of the chalkboard, PS, and ongoing intention to utilize the blackboard are all dependent constructs. Multiple items were used to assess each of the components. To maintain secrecy, the questionnaire was circulated online using Google Forms, emails, and WhatsApp, and responders’ personal information was then destroyed.

### Design of the study

This study employed a quantitative approach and used a cross-sectional survey (Fraenkel et al., 2011). This method was chosen because it has a reputation for providing reliable, valid, and generalizable findings (Fraenkel et al., 2011). Furthermore, a questioner survey might be sent to a large number of people. A quantitative study also helps researchers to make sweeping generalizations about a community when data is collected from a representative sample (Fraenkel et al., 2011). A quantitative investigation (Hair et al., 2017), requires a sufficient sample size. Various rules for determining an appropriate sample size for regression analysis have been proposed. The ‘10-time rules (Hair et al., 2017), are the most widely used large sample estimation technique in PLS-SEM, implying that sample size ought to be equal to the greater values between both the conceptual framework with the most formative indicators and the intrinsic construct with more impartial exogenous construct trying to predict it. The endogenous construct is a construct that is created by the body itself. In terms of sample size, the survey had 159 replies, which is a satisfactory sample size. Furthermore, a questioner survey was employed in this study since it is one of the most extensively used methodologies in technology acceptance (Lew et al., 2019). Furthermore, because of its convenience and accessibility across numerous platforms, this study performed an online survey (Fraenkel et al., 2011). As the participants were studying from home, they were contacted *via* the e-learning system’s chat function and a WhatsApp group. Participants were provided a link to a Google Forms-hosted questionnaire, which remained accessible for 3 weeks. The suggested model was utilized to test the assumptions using structural equation modeling (SEM). PLS-SEM (partial least squares SEM) is an appropriate solution for this study’s goal. As a result, SMART PLS 2.0 was used in this study to assess confirmatory factor analysis (CFA) and confirm the model’s reliability, validity, and internal consistency.

### Pre-test and pilot test

Pre-tests allow for the consideration of issues that cannot be expected during the administration of the questionnaire, assisting the researcher in obtaining better findings. Meanwhile, pilot testing tries to determine whether the instrument will operate as a real—time projects by implementing it with a small—scale pilot group and finding any flaws in the questions prior to a field launch. Initially, the respondents were issued 20 questionnaires, and the exploratory factor analysis revealed that each of the eleven factors was reliable and legitimate. A few small problems expressed during the pilot research were addressed, including the clarity of the instructions and questions, the overall design, as well as other minor observations. To ensure that the scales are meaningful, all ambiguities were eliminated.

### Instrument measurement

The questionnaire scales were derived from previously verified surveys. Tselios et al. (2011) and Venkatesh et al. (2003) provided the four items of PE, while (Wang and Shih, 2009) provided the five items of EE. The four questions measuring SI were adopted from Taylor and Todd (1995), whereas the six items measuring continuing intention were adapted from Lin and Wang (2012) and Venkatesh et al. (2003). Five questions were modified from each of the enabling circumstances and behavioral intention categories (Venkatesh et al., 2003). Furthermore, four items were altered for each aspect from the perceived value, usefulness and ease of use, user satisfaction, and actual usage blackboard (Davis, 1989). Finally, five satisfaction-related items were selected (Lee, 2010a).

## Results and analysis

The partial least square structural equation modeling (PLS-SEM) approach was used to analyze the data in this study, and Smart PLS version 2.0 was used (Ringle et al., 2005). PLS-SEM was carried out in two stages. The first phase is evaluating the measurement model, while the second entails evaluating the structural model. The concept validity and discriminant validity criteria were tested in the measurement model, while the R2 and significance of the path coefficients were assessed in the structural model.

### Demographics data

As shown in Table 1, the demographic analysis revealed the following description of the respondent profiles. The gender breakdown of the respondents revealed that female respondents made up 69.8% of the total, with male respondents accounting for 30.2 percent. The investigation revealed that 10.7% of the respondents were 18–21 years old, 15.1% were 22–25 years old, 22.0% were 26–29 years old, 28.3% were 30–33 years old, and 23.9% were older than 34 years old. Furthermore, the investigation revealed that 13.8 percent had a bachelor’s degree, 44.0 percent had a master’s degree, and 42.1 percent had a Ph.D. degree. Furthermore, the data revealed that 8.8% of those who used a chalkboard for less than 1 year, 17.0% of those who used a blackboard for 1 year, 34.6 percent of those who used a blackboard for 2 years, and 39.6% of those who used a blackboard for more than 2 years. Finally, 18.9% of universities were from KSU, 19.5 percent from KAU, 20.1 percent from KFU, 21.4 percent from PNU, and 20.1 percent from UT, according to the data.

**TABLE 1:** Demographic analysis.

Gender	Frequency	Percent	Level of education	Frequency	Percent
Male	48	30.2	Bachelor	22	13.8
Female	111	69.8	Master	70	44.0
Total	159	100.0	Ph.D.	67	42.1
**Age**	**Frequency**	**Percent**	Total	159	100.0
18–21	17	10.7	**Universities**	**Frequency**	**Percent**
22–25	24	15.1	KSU	30	18.9
26–29	35	22.0	KAU	31	19.5
30–33	45	28.3	KFU	32	20.1
>34	38	23.9	PNU	34	21.4
Total	159	100.0	UT	32	20.1
**Time of Use**	**Frequency**	**Percent**	Total	159	100.0
<1 Year	14	8.8	**Time of Use**	**Frequency**	**Percent**
1 Year	27	17.0	More 2 Years	63	39.6
2 Years	55	34.6	Total	159	100.0

### Measurement model evaluation

Construct validity refers to how well a test examines everything it needs to measure. Construct validity, validities, and criteria validity are the three basic types of validated evidence (Hair et al., 2019). According to factor analysis, factors have a lot of item load and pass (Table 2).

**TABLE 2:** Factors loadings and cross-loadings of items.

Factors	Items	AUB	BIU	ICU	EE	FC	PS	PE	PEU	PU	SI	PEX
**Actual Use of Blackboard**	**AUB1**	0.944	0.848	0.868	0.747	0.687	0.852	0.788	0.821	0.789	0.580	0.774
	**AUB2**	0.936	0.802	0.838	0.715	0.669	0.859	0.696	0.730	0.729	0.556	0.719
	**AUB3**	0.954	0.801	0.864	0.735	0.711	0.866	0.736	0.761	0.763	0.579	0.759
	**AUB4**	0.929	0.806	0.827	0.749	0.726	0.834	0.785	0.793	0.719	0.596	0.793
**Behavioral Intention Use**	**BIU1**	0.822	0.935	0.802	0.777	0.743	0.869	0.821	0.819	0.829	0.597	0.779
	**BIU2**	0.848	0.956	0.771	0.751	0.700	0.855	0.835	0.801	0.788	0.567	0.767
	**BIU3**	0.807	0.954	0.748	0.740	0.706	0.810	0.829	0.795	0.772	0.559	0.783
	**BIU4**	0.822	0.965	0.791	0.785	0.749	0.844	0.853	0.840	0.796	0.637	0.814
**Intention to Continue Using**	**ICU1**	0.775	0.701	0.898	0.683	0.676	0.712	0.639	0.732	0.723	0.565	0.682
	**ICU2**	0.879	0.777	0.946	0.695	0.695	0.832	0.729	0.795	0.733	0.593	0.716
	**ICU3**	0.881	0.800	0.961	0.719	0.679	0.849	0.721	0.799	0.766	0.562	0.744
	**ICU4**	0.837	0.773	0.935	0.763	0.693	0.786	0.693	0.759	0.814	0.585	0.746
**Effort Expectancy**	**EE1**	0.700	0.718	0.695	0.947	0.734	0.702	0.735	0.764	0.778	0.560	0.741
	**EE2**	0.729	0.756	0.713	0.964	0.768	0.746	0.800	0.813	0.799	0.605	0.793
	**EE3**	0.751	0.758	0.735	0.945	0.762	0.717	0.776	0.821	0.798	0.626	0.777
	**EE4**	0.768	0.785	0.733	0.911	0.740	0.775	0.792	0.816	0.788	0.604	0.816
**Facilitating Conditions**	**FC1**	0.703	0.679	0.684	0.780	0.906	0.696	0.689	0.717	0.707	0.633	0.709
	**FC2**	0.738	0.742	0.721	0.713	0.918	0.742	0.731	0.702	0.651	0.696	0.739
	**FC3**	0.669	0.721	0.662	0.675	0.905	0.690	0.702	0.680	0.651	0.733	0.705
	**FC4**	0.502	0.542	0.516	0.655	0.811	0.521	0.544	0.579	0.581	0.754	0.530
**Perceived Satisfaction**	**PS1**	0.919	0.859	0.841	0.737	0.721	0.947	0.757	0.777	0.771	0.563	0.743
	**PS2**	0.861	0.846	0.811	0.745	0.715	0.959	0.802	0.781	0.786	0.589	0.802
	**PS3**	0.824	0.814	0.779	0.748	0.705	0.947	0.789	0.763	0.817	0.578	0.774
	**PS4**	0.846	0.857	0.812	0.743	0.722	0.955	0.807	0.783	0.780	0.588	0.784
**Perceived Enjoyment**	**PE1**	0.769	0.826	0.716	0.793	0.703	0.775	0.946	0.843	0.742	0.582	0.823
	**PE2**	0.793	0.861	0.745	0.785	0.757	0.826	0.963	0.830	0.745	0.640	0.874
	**PE3**	0.717	0.813	0.663	0.775	0.697	0.763	0.946	0.783	0.731	0.563	0.841
**Perceived Ease of Use**	**PEU1**	0.723	0.727	0.700	0.813	0.682	0.720	0.772	0.899	0.782	0.538	0.744
	**PEU2**	0.799	0.820	0.786	0.762	0.662	0.777	0.777	0.925	0.791	0.602	0.759
	**PEU3**	0.814	0.818	0.839	0.764	0.711	0.792	0.800	0.930	0.799	0.610	0.751
	**PEU4**	0.695	0.784	0.689	0.733	0.662	0.703	0.791	0.890	0.712	0.569	0.766
	**PEU5**	0.696	0.709	0.710	0.789	0.713	0.688	0.752	0.875	0.754	0.531	0.691
**Perceived Usefulness**	**PU1**	0.766	0.780	0.780	0.794	0.722	0.816	0.753	0.808	0.885	0.557	0.732
	**PU2**	0.735	0.733	0.731	0.764	0.677	0.728	0.669	0.724	0.918	0.491	0.694
	**PU3**	0.746	0.746	0.746	0.776	0.639	0.746	0.682	0.772	0.937	0.503	0.701
	**PU4**	0.684	0.774	0.699	0.757	0.642	0.722	0.704	0.781	0.929	0.532	0.726
	**PU5**	0.733	0.805	0.767	0.767	0.685	0.788	0.754	0.812	0.926	0.567	0.746
**Superior Influence**	**SI1**	0.497	0.530	0.532	0.527	0.730	0.500	0.523	0.543	0.484	0.907	0.557
	**SI2**	0.514	0.514	0.521	0.528	0.713	0.500	0.496	0.517	0.488	0.918	0.539
	**SI3**	0.639	0.635	0.633	0.655	0.697	0.656	0.672	0.648	0.593	0.865	0.687
	**SI4**	0.545	0.537	0.514	0.565	0.706	0.513	0.538	0.549	0.499	0.919	0.609
**Performance Expectancy**	**PEX1**	0.788	0.834	0.747	0.794	0.724	0.817	0.902	0.790	0.775	0.616	0.940
	**PEX2**	0.761	0.757	0.720	0.807	0.702	0.753	0.835	0.807	0.725	0.629	0.935
	**PEX3**	0.703	0.701	0.683	0.709	0.699	0.695	0.731	0.684	0.681	0.623	0.913

The research approach for this study included 44 indicator items and 11 aspects of student happiness and continuous use characteristics such as PS and desire to continue using Blackboard. The measurement model was evaluated using Cronbach’s reliability, composite reliability, and convergence validity tests (Hair et al., 2017). Cronbach’s reliability ratings for all latent variables were substantially higher than the minimum appropriate standard of 0.4 and near to the ideal level of 0.7 (Table 3). The convergent validity was likewise more than 0.7, indicating that all eleven reflecting latent constructs had excellent measure of internal consistency dependability. In addition, the average variance extracted (AVE) for each latent variable was tested to ensure convergent validity. Because all of the AVE values in the measurement items above the permissible threshold of 0.5, convergent validity was established (Table 3).

**TABLE 3:** Confirmatory factor analysis.

Factors	Items	Factors loadings	Composite reliability	Cronbach’s alpha	AVE	*R* square
**Actual Use of Blackboard**	**AUB1**	0.944	0.968	0.956	0.884	0.777
	**AUB2**	0.936				
	**AUB3**	0.954				
	**AUB4**	0.929				
**Behavioral Intention Use**	**BIU1**	0.935	0.975	0.966	0.907	0.774
	**BIU2**	0.956				
	**BIU3**	0.954				
	**BIU4**	0.965				
**Intention to Continue Using**	**ICU1**	0.898	0.965	0.952	0.874	0.823
	**ICU2**	0.946				
	**ICU3**	0.961				
	**ICU4**	0.935				
**Effort Expectancy**	**EE1**	0.947	0.969	0.957	0.886	0.000
	**EE2**	0.964				
	**EE3**	0.945				
	**EE4**	0.911				
**Facilitating Conditions**	**FC1**	0.906	0.935	0.908	0.785	0.000
	**FC2**	0.918				
	**FC3**	0.905				
	**FC4**	0.811				
**Perceived Satisfaction**	**PS1**	0.947	0.974	0.965	0.906	0.000
	**PS2**	0.959				
	**PS3**	0.947				
	**PS4**	0.955				
**Perceived Enjoyment**	**PE1**	0.946	0.966	0.947	0.905	0.811
	**PE2**	0.963				
	**PE3**	0.946				
**Perceived Ease of Use**	**PEU1**	0.899	0.957	0.944	0.817	0.809
	**PEU2**	0.925				
	**PEU3**	0.930				
	**PEU4**	0.890				
	**PEU5**	0.875				
**Perceived Usefulness**	**PU1**	0.885	0.964	0.954	0.844	0.777
	**PU2**	0.918				
	**PU3**	0.937				
	**PU4**	0.929				
	**PU5**	0.926				
**Superior Influence**	**SI1**	0.907	0.946	0.924	0.814	0.000
	**SI2**	0.918				
	**SI3**	0.865				
	**SI4**	0.919				
**Performance Expectancy**	**PEX1**	0.940	0.950	0.921	0.863	0.000
	**PEX2**	0.935				
	**PEX3**	0.913				

As stated in Table 4, the purpose of construct is to see if latent variables differ from one another by analyzing within both correlation with the actual figures of their relative reference variances obtained. The square root of the AVEs for each latent construct must be greater than the correlation for that latent variable when assessing the square roots of the AVEs to the other values in each column (Hair et al., 2017). According to Hair et al. (2017), the outer weight reflects how much each signal impacts the latent variables.

**TABLE 4:** Discriminant validity.

No.	Factors	1	2	3	4	5	6	7	8	9	10	11
1	**Actual Use of Blackboard**	0.917										
2	**Behavioral Intention**	0.866	0.890									
3	**Effort Expectancy**	0.783	0.802	0.883								
4	**Facilitating Conditions**	0.742	0.761	0.798	0.877							
5	**Intention to Continue Using**	0.903	0.817	0.764	0.733	0.863						
6	**Perceived Ease of Use**	0.826	0.855	0.854	0.758	0.826	0.940					
7	**Perceived Enjoyment**	0.799	0.876	0.824	0.756	0.745	0.861	0.912				
8	**Perceived Satisfaction**	0.907	0.887	0.781	0.752	0.852	0.815	0.828	0.900			
9	**Perceived Usefulness**	0.798	0.836	0.840	0.733	0.811	0.850	0.777	0.828	0.897		
10	**Performance Expectancy**	0.809	0.825	0.830	0.762	0.772	0.821	0.889	0.815	0.784	0.927	
11	**Superior Influence**	0.614	0.620	0.637	0.790	0.615	0.631	0.625	0.609	0.578	0.670	0.932

### Structural model evaluation

**FIGURE 2:**
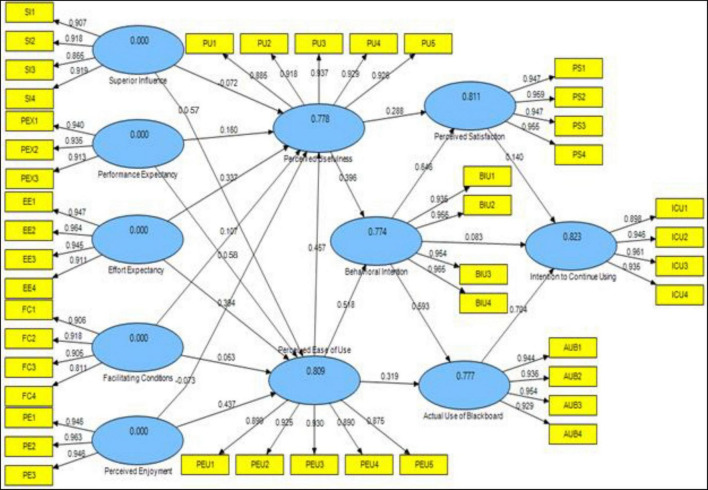
Research model.

To determine the statistically significant of each hypothesis, the structural equation model was reviewed and inspected using the both probability value and path coefficient numbers (Hair et al., 2017). The *R*-Square values were PU (0.778), PEU (0.809), behavioral intention (0.774), AUB (0.777), PS (0.811), and intention to continue using (0.823), as shown in Table 3 and Figure 2. This means that the six latent variables explained 82.3 percent of the variance in lecturers’ intention to continue using blackboard at Saudi universities.

**FIGURE 3:**
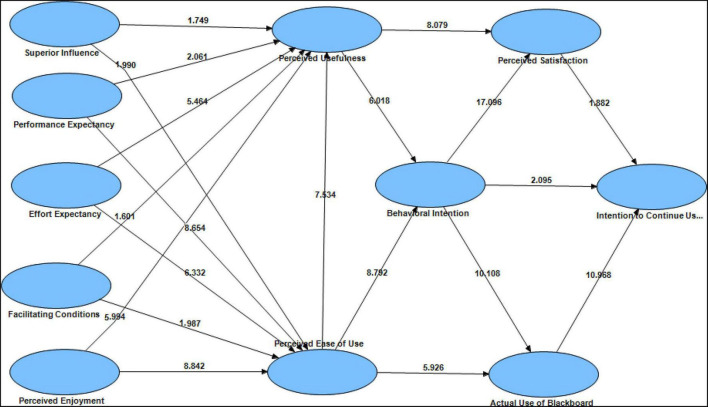
Research model with *t*-values.

The findings of the route ratings and *p*-values for all of the proposed hypotheses are shown in Figure 3 and Table 5. The importance of the predicted relationships linking the constructs is determined by the route coefficients; twenty hypotheses were accepted because the *t*-values were larger than 1.5. While real blackboard use was hypothesized to have a substantial effect on intention to keep using blackboard during the COVID-19 outbreak, the findings emphasized all assumptions accepted. The complete data and structural model are shown in Figure 3.

**TABLE 5:** Hypotheses testing.

Hypotheses relationships	Path	Mean	St.D	*T*-values	Results
Superior Influence- > Perceived Usefulness (H1)	0.072	0.071	0.042	1.749	Accepted
Superior Influence- > Perceived Ease of Use (H2)	0.04	0.037	0.027	1.99	Accepted
Performance Expectancy- > Perceived Usefulness (H3)	0.16	0.157	0.075	2.061	Accepted
Performance Expectancy- > Perceived Ease of Use (H4)	0.038	0.04	0.058	8.654	Accepted
Effort Expectancy- > Perceived Usefulness (H5)	0.337	0.329	0.06	5.464	Accepted
Effort Expectancy- > Perceived Ease of Use (H6)	0.394	0.389	0.058	6.332	Accepted
Facilitating Conditions- > Perceived Usefulness (H7)	0.107	0.114	0.067	1.601	Accepted
Facilitating Conditions- > Perceived Ease of Use (H8)	0.053	0.059	0.05	1.987	Accepted
Perceived Enjoyment- > Perceived Usefulness (H9)	0.073	−0.065	0.072	5.994	Accepted
Perceived Enjoyment- > Perceived Ease of Use (H10)	0.437	0.437	0.048	8.842	Accepted
Perceived Ease of Use- > Perceived Usefulness (H11)	0.457	0.453	0.06	7.534	Accepted
Perceived Ease of Use- > Behavioral Intention (H12)	0.518	0.52	0.059	8.792	Accepted
Perceived Ease of Use- > Actual Use of Blackboard (H13)	0.319	0.321	0.055	5.926	Accepted
Perceived Usefulness- > Perceived Satisfaction (H14)	0.288	0.286	0.035	8.079	Accepted
Perceived Usefulness- > Behavioral Intention (H15)	0.396	0.394	0.065	6.018	Accepted
Behavioral Intention- > Actual Use of Blackboard (H16)	0.593	0.591	0.058	10.108	Accepted
Behavioral Intention- > Perceived Satisfaction (H17)	0.646	0.647	0.036	17.096	Accepted
Behavioral Intention- > Intention to Continue Using (H18)	0.083	0.084	0.074	2.095	Accepted
Perceived Satisfaction- > Intention to Continue Using (H19)	0.14	0.139	0.072	1.882	Accepted
Actual Use of Blackboard- > Intention to Continue Using (H20)	0.704	0.704	0.059	10.968	Accepted

Table 5 showed the 20 hypotheses relationships between the eleven factors that developed a new model to measure art education lecturers’ intention to continue using the blackboard during and after the COVID-19 pandemic through an empirical investigation into the UTAUT and TAM model. The relationship between SI and PU (Path = 0.072, *t*-value = 1.749), H1 accepted. Similarly, the relationship between SI and PU (Path = 0.057, *t*-value = 1.99), H2 accepted. Next the relationship between PE and PU (Path = 0.160, *t*-value = 2.061), H3 accepted. Similarly, the relationship between PE and PU (Path = 0.058, *t*-value = 8.654), H4 accepted. Besides, the relationship between EE and PU (Path = 0.337, *t*-value = 5.464), H5 accepted. Similarly, the relationship between EE and PU (Path = 0.394, *t*-value = 6.332), H6 accepted. In addition, the relationship between FC and PU (Path = 0.107, *t*-value = 1.601), H7 accepted. Similarly, the relationship between FC and PU (Path = 0.053, *t*-value = 1.987), H8 accepted. Moreover, the relationship between PE and PU (Path = 0.073, *t*-value = 5.994), H9 accepted. Similarly, the relationship between PE and PU (Path = 0.437, *t*-value = 8.842), H10 accepted. Furthermore, the relationship between PEU and PU (Path = 0.457, *t*-value = 7.534), H11 accepted. Similarly, the relationship between PEU and behavioral intention (Path = 0.518, *t*-value = 8.792), H12 accepted. And the relationship between PEU and AUB (Path = 0.319, *t*-value = 5.926), H13 accepted. Additionally, the relationship between PU and PS (Path = 0.288, *t*-value = 8.079), H14 accepted. Similarly, the relationship between PU and behavioral intention (Path = 0.396, *t*-value = 6.018), H15 accepted. As well, the relationship between behavioral intention and AUB (Path = 0.593, *t*-value = 10.108), H16 accepted. Similarly, the relationship between behavioral intention and PS (Path = 0.646, *t*-value = 17.096), H17 accepted. And the relationship between behavioral intention and intention to continue using blackboard (Path = 0.083, *t*-value = 2.095), H18 accepted. Finally, the relationship between PS and intention to continue using blackboard (Path = 0.140, *t*-value = 1.882), H19 accepted. Similarly, the relationship between AUB and intention to continue using blackboard (Path = 0.704, *t*-value = 10.968), H20 accepted.

### Factors described and analyzed

**FIGURE 4:**
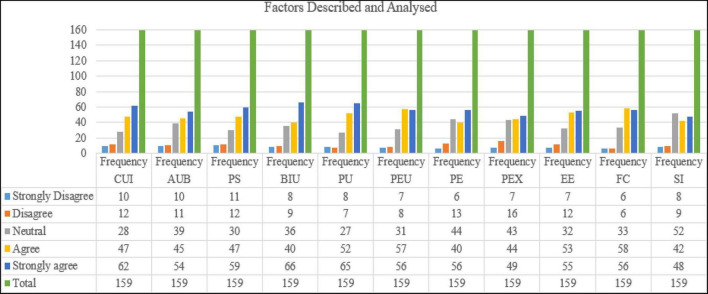
Factors described and analyzed.

The standard deviation (SD) and mean (mean) are two statistics that show how measurements differ from the average (mean) or anticipated value in a population. The bulk of data points are near to the mean when the standard deviation is low. If the standard deviation is large, the data is more evenly dispersed. As a consequence, as shown in Figure 4, all values were accepted and the majority was agreed and strongly agreed, meaning that the critical factors that influence students’ actual use of blended learning in higher education through self-directed learning, students’ self-efficacy, motivation to learn, learning control, learning autonomy, students’ readiness, perceived behavioral control, students’ attitude toward use, behavioral intention to use, and actual use of blended learning, see Figure 4.

## Discussion

This study’s findings showed a number of consequences. The first is an expansion of the UTAUT theory and TAM model to make it relevant to the present pandemic scenario, as well as its application in higher education to look at the acceptability of online learning systems. Saudi Arabian universities will focus on boosting professor success by upgrading the interface and introducing learning management systems such as blackboard functionality. As a result, this study contributes to an expanded model of UTAUT and TAM, in which the incorporation of pleasure reinforces and drives lecturers’ intentions to use Blackboard in the future. Furthermore, the conventional education system in Saudi Arabia strives to contribute value where research is lacking. The results of this study might help university administrators analyze the aspects that influence art education professors’ happiness with Blackboard in the long run. Furthermore, the findings might be applied to comparable situations, particularly following the implementation of new information systems, whether they are utilized by students or instructors.

The found multiple model indices were met and acceptable, as shown in Figure 2. As a result, the researcher arrived at the structural equation model shown on the preceding page. The enabling condition had the greatest coefficient among the four variables in the UTAUT model, indicating that it is the strongest predictor of lecturers’ behavioral intention to utilize Blackboard. The enabling condition (Venkatesh et al., 2012), refers to users’ perceptions of the resources and assistance available to complete an action. As a result of this study, it was discovered that the facilitating condition had a beneficial influence on PEU and usefulness, which in turn had a good impact on behavioral intention and actual usage of Blackboard. It was discovered that the technology’s performance is critical to lecturers’ perceptions, which has a favorable influence on satisfaction and continued usage of Blackboard. Other research have found similar results (Šumak et al., 2010; El-Masri and Tarhini, 2017; Siswanto et al., 2018). Others, on the other hand, disagree (Mallat et al., 2008). Furthermore, PE was found to have a beneficial influence on PEU and usefulness, which in turn had a positive impact on behavioral intention and actual usage of Blackboard. It was discovered that the technology’s performance is critical to lecturers’ perceptions, which has a favorable influence on satisfaction and continued usage of Blackboard. This discovery is consistent with findings from other research, including as Abu-Al-Aish and Love (2013), Alshehri et al. (2020), Sultana (2020), and Raza et al. (2021). Others, such as Šumak et al. (2010), disagree (2010). Students and professors will embrace the Blackboard platform once they see how well it performs. Furthermore, a rise in lecturers’ happiness with Blackboard only stimulates the usage of Blackboard indefinitely. It has the potential to improve Saudi universities’ online learning and Blackboard systems. However, this result contradicted (Kolog et al., 2015). In terms of the moderating variables that impact the link between performance expectation, EE, social influence, and facilitating condition and instructors’ intentions to use Blackboard.

Another study was that effort anticipation had a substantial and favorable impact on PEU and utility, which in turn influenced behavioral intention and actual usage of Blackboard. It was discovered that the technology’s performance is critical to lecturers’ perceptions, which has a favorable influence on satisfaction and continued usage of Blackboard. This conclusion is consistent with findings from previous investigations (Abu-Al-Aish and Love, 2013; Bouznif, 2018; Sultana, 2020; Mujalli et al., 2022). Nonetheless, whether effort expectation is an important predictor of behavioral intention, which leads to higher use of Blackboard, is debatable. Furthermore, a rise in lecturers’ happiness with Blackboard only stimulates the usage of Blackboard indefinitely.

According to the findings of this study, SI had a big and positive impact on PEU and usefulness, which in turn had a positive impact on behavioral intention and AUB. SI, a type of social influence, was used to reveal a difficult issue. In fact, additional studies (Bellaaj et al., 2015) concurred with our result that social impact had no effect on the intention to continue using. Furthermore, in both (Taylor and Todd, 1995; Lee, 2010b) research, subjective norms (social influence) were found to have a significant impact on continuing intention. Despite this, Lwoga and Komba (2015) discovered that instructors’ views toward using Blackboard had a substantial influence on students’ purposeful activities.

Perceived pleasure had a large and favorable influence on PEU and usefulness, which in turn had a good impact on behavioral intention and actual usage of Blackboard, according to this study’s findings. As teaching and learning shifts from face-to-face to totally online, having an easy-to-use Blackboard that allows lecturers and students to learn independently will undoubtedly result in more engaging teaching experiences and enhanced teaching and learning (Han and Shin, 2016). It is vital for education, particularly for students from underprivileged backgrounds, to develop ways to make the use of technology fun in order to fully benefit from learning management systems such as Blackboard (Matarirano et al., 2021). As a result, this research shows that using Blackboard as an online LMS can have a favorable impact on lecturers’ performance during the epidemic, resulting in more engaging teaching experiences.

Perceived simplicity of use and PU were found to have a substantial and favorable influence on behavioral intention, satisfaction, and actual usage of Blackboard in this study. According to Alkhaldi and Abualkishik (2019), the respondents believed that blackboard is simple to use, and that effort expectation has a good influence on the intention to use blackboard. This conclusion is consistent with findings from previous research, such as Capuchino et al. (2020), which found that respondents highly agreed that blackboard open LMS are effective in their classrooms. In addition, the respondents, who are King Saud University instructors, agreed that the Blackboard e-learning program is simple to access and utilize (Alturki and Aldraiweesh, 2016). Overall, this research backs up TAM, since PEU was found to be a strong predictor of academic success. This conclusion backs with prior research by Al-Naibi (2016), who found that in order to increase users’ knowledge of blackboard’s many features, blackboard developers should give more training to consumers or create solution systems when there are no apparent plugins (Vrielink, 2015). Users who believe the system is simple to use are more likely to utilize it in the future, which improves their academic achievement (Abdullah et al., 2019) as well as their desire to use it (Ismail, 2016). Furthermore, according to this research, how instructors use the vast array of functions offered on Blackboard becomes a future trend. As a result, the findings of this study can serve as a guide for blackboard developers, who should consider future training for professors and students in order to increase users’ PEU and efficiency. Finally, this research finds that blackboard, as an internet Blackboard, has a good influence on teaching during in the COVID-19 epidemic. Furthermore, Daneji et al. (2017) discovered that perceived utility can increase the likelihood of using Blackboard platforms. As a result of this research, university administrators and professors may boost blackboard’s PU by encouraging students to utilize it. Teachers can push students to utilize blackboard to get higher results (Dulkaman and Mohamad Ali, 2016). Students will find blackboard more useful if their grades rise, which will boost blackboard’s PU. The new educational system has had an influence on the United Nations’ Sustainable Development Goals, according to a recent systematic review, with an increased risk of sustainability in tertiary education (Crawford and Cifuentes-Faura, 2022). Students, on the other hand, received insufficient social support and security protection from others and their teachers when they needed it (Cifuentes-Faura et al., 2021). Furthermore, universities face difficulties in maintaining consistency and relevance in course content, communicating clearly with the academic community, and acquiring and recruiting students (Marinoni et al., 2020), as well as the concept of supporting evidence-based practices to promote scholarly teaching practices (García-Morales et al., 2021). As a result, in order to bridge the digital divide and encourage sustainable activities, higher education institutions must ensure that education is inclusive, equitable, and of high quality (Faura-Martínez et al., 2021). Furthermore, this study supports both behavioral intent and actual usage of the chalkboard (Davis, 1989; Wang and Wang, 2009; Motaghian et al., 2013; Raman and Don, 2013) all came to similar conclusions. They came to the conclusion that there is a strong correlation among behavioral intentions and behavior chalkboard use. His or her behavioral control refers to a person’s intention to embrace the usage of a given technology for various activities (Ain et al., 2016). Muniasamy et al. (2014) employed the TAM to evaluate the correlations between PU and PEU, PU, and behavioral control to use online learning in Saudi Arabia’s King Khalid University. According to Binyamin et al. (2018) used the TAM model to determine the use of blackboard among university students at King Abdulaziz University in Saudi Arabia. Similarly, Binyamin et al. (2019) used TAM to investigate the characteristics that influence students’ use of Blackboard at King Abdulaziz University in Saudi Arabia. Other studies have looked into the factors that influence faculty members’ willingness to use e-learning systems using TAM, including (Al Meajel and Sharadgah, 2018)’s study of the blackboard system at King Saud University, Ahmed (2016) in Najran University in Saudi Arabia, and (Alsuwailem, 2018) about online learning at King Faisal University in Saudi Arabia, and (Alharbi and Drew, 2014) study of LMS such as Blackboard at Shaqra University. They’ve all validated the TAM fundamental elements’ considerable impact and linkage (PEU, PU with behavioral intention, and actual use blackboard). As a result, the authors of this study predict a positive relationship between the Learning Management System’s behavioral intention and its lecturer’s behavior intention and actual usage of the blackboard, which is consistent with the current literature. Finally, during the COVID-19 outbreak, this study looked into the factors that determined lecturers’ happiness with blackboard usage in higher education. The UTAUT model precisely suited the effect of pleasure as a variable on continuing intention. Prior study (Alharbi and Drew, 2014; Weng et al., 2015) found that satisfaction was a strong predictor of continuing usage intention in an enlarged TAM model (Alharbi and Drew, 2014; Weng et al., 2015). As a consequence, it is possible to derive the crucial and influential role of satisfaction as a mediation and an outcome variable in determining continuing intention. The survey also found that instructors’ satisfaction has a substantial influence on their continued desire to utilize blackboard. However, the findings demonstrated a considerable impact of satisfaction on the long-term intention to use the chalkboard throughout the epidemic, which is supported by other recent research (Alzahrani and Seth, 2021). Furthermore, the study discovered that instructors’ pleasure influenced their continued purpose to use blackboard throughout and after the epidemic. In conclusion, the following are the research contributions:

• Incorporating Blackboard into educational strategies can improve students’ Behavioral Intention to use it for digital learning.
• Lecturers and supervisors should encourage students to use Blackboard to solve problems, share knowledge, and provide information in order to enhance their learning experiences, success, and research skills.
• It is recommended that higher education institutions recognize students who are comfortable with using Blackboard in the classroom rather than pressuring someone who is not familiar to do so. This is due to the fact that students must incorporate Blackboard components and resources into their learning process.
• Students’ attitudes about using Blackboard for digital learning, as well as their intentions to use Blackboard for digital learning, are concerned with both technology and resources. Students should take advantage of opportunities to use Blackboard for digital learning.

However, of the viewpoints it presents, this research has its own limitations. To begin with, because this study only looked at one university, its findings should be interpreted with caution, as activity at other universities (private institutions and other universities) may differ. Another disadvantage is that this analysis relies on quantitative data; as a result, the researchers should employ a qualitative data technique (interviews or observations) to prevent overlooking disparities across research fields. To solve its flaws and widen its results, future study should re-create this analysis in different settings, countries, and cultures.

### Conclusion, limitations and future research

During and after the COVID-19 epidemic, this study studied and examined the crucial aspects determining lecturers’ satisfaction and continued usage of blackboard in Saudi higher education. An online poll was used to suggest and verify a structural research model. PE, EE, SI, enabling circumstances, and reported enjoyment all had a substantial impact on PU and PEU throughout the epidemic, according to the findings. Furthermore, the data revealed that perceived utility and perceived simplicity of use had a substantial impact on lecturers’ behavioral intention, as well as actual usage of blackboard for teaching art education in Saudi universities. Furthermore, lecturers’ behavioral intention and actual usage of blackboard during and after the COVID-19 outbreak influenced lecturers’ satisfaction and their desire to continue using blackboard in higher education.

The findings have important implications for educators, policymakers, and practitioners who want to design and improve successful ways for using chalkboard during and after COVID-19. The study, however, contains three flaws. To increase the generalizability of the results, the data were obtained from a limited sample of Saudi public institutions; consequently, the scope of universities and the quantity of lecturers and students in universities who use the blackboard system should be expanded. Furthermore, the study employed quantitative research methods, whereas qualitative evaluation might offer more reasons for the hypothesized constructs’ correlations. As a result, future research should include a qualitative method to supplement quantitative findings. Finally, further cross-sectional and cross-cultural research is needed to improve the predictive value of blackboard usage in higher education in other countries. Furthermore, the authors recommend that the extended UTAUT theory and TAM model be investigated in other developed and developing countries during and after the pandemic, that factors influencing blackboard acceptance and use of online learning and mobile learning systems be investigated, and that better course material and assistance be provided to students in pursuit of education, whether it is used in blackboard in arts education classes or other fields of education, be provided. Aside from that, mediator and moderator factors may be added to the model to further extend it and analyze processes that are relevant to the present scenario.

## Data availability statement

The original contributions presented in this study are included in the article/supplementary material, further inquiries can be directed to the corresponding author.

## Author contributions

The author confirms being the sole contributor of this work and has approved it for publication.
